# QTL Mapping for Fiber Quality and Yield Traits Based on Introgression Lines Derived from *Gossypium hirsutum* × *G. tomentosum*

**DOI:** 10.3390/ijms19010243

**Published:** 2018-01-14

**Authors:** Ayaz Ali Keerio, Chao Shen, Yichun Nie, Muhammad Mahmood Ahmed, Xianlong Zhang, Zhongxu Lin

**Affiliations:** National Key Laboratory of Crop Genetic Improvement, College of Plant Science and Technology, Huazhong Agricultural University, Wuhan 430070, China; ayazalikeerio@outlook.com (A.A.K.); shen@webmail.hzau.edu.cn (C.S.); nyc@mail.hzau.edu.cn (Y.N.); mhemud@hotmail.com (M.M.A.); xlzhang@mail.hzau.edu.cn (X.Z.)

**Keywords:** Upland cotton, *Gossypium tomentosum*, introgression lines, QTL mapping, fiber quality traits, yield

## Abstract

The tetraploid species *Gossypium hirsutum* is cultivated widely throughout the world with high yield and moderate fiber quality, but its genetic basis is narrow. A set of 107 introgression lines (ILs) was developed with an interspecific cross using *G. hirsutum* *acc*. 4105 as the recurrent parent and *G. tomentosum* as the donor parent. A specific locus amplified fragment sequencing (SLAF-seq) strategy was used to obtain high-throughput single nucleotide polymorphism (SNP) markers. In total, 3157 high-quality SNP markers were obtained and further used for identification of quantitative trait loci (QTLs) for fiber quality and yield traits evaluated in multiple environments. In total, 74 QTLs were detected that were associated with five fiber quality traits (30 QTLs) and eight yield traits (44 QTLs), with 2.02–30.15% of the phenotypic variance explained (PVE), and 69 markers were found to be associated with these thirteen traits. Eleven chromosomes in the A sub-genome (At) harbored 47 QTLs, and nine chromosomes in the D sub-genome (Dt) harbored 27 QTLs. More than half (44 QTLs = 59.45%) showed positive additive effects for fiber and yield traits. Five QTL clusters were identified, with three in the At, comprised of thirteen QTLs, and two in the Dt comprised of seven QTLs. The ILs developed in this study and the identified QTLs will facilitate further molecular breeding for improvement of Upland cotton in terms of higher yield with enhanced fiber quality.

## 1. Introduction

Cotton is one of the cash crops around the world that provides superior fiber for the textile industry. Among the four cotton cultivated species, Upland cotton (*Gossypium hirsutum*) produces 95% of the world’s cotton and is characterized as high yield with moderate fiber quality and wide adaptability [1,2]. In the last few years, worldwide cotton cultivation has declined, mainly due to high production costs and strong market competition with other crops [3]. Upland cotton dominates world cotton production with its high yield, but its fiber quality is undesirable, whereas Sea Island cotton (*G. barbadense*) is known to have the best fiber quality but a lower yield. Consequently, one of the goals of world-wide cotton breeding programs is improving Upland cotton cultivars.

With the globally increasing demand for textile products and strong competition from synthetic fibers, the need for Upland cotton cultivars with high yield and improved fiber has never been more critical [4]. Both fiber quality and yield are complex traits that are controlled by a magnitude of QTLs [5]. There is a complicated genetic correlation among fiber and yield traits because of the different population types and parental lines [6,7,8,9]. Thus, improving both fiber quality and yield simultaneously is a long-term task for cotton breeders. Conventional breeding procedures are becoming increasingly difficult because of their long duration and low selection efficiency [10].

The presence of genetic variability in cultivated plants for any breeding program is sometimes restricted, particularly when gene pools are not easily accessible. Wild related cotton species have long been used as genetic sources for incorporating new desirable traits to enhance the potential of cotton cultivars [11]. Three allotetraploid cotton species that are known to exist only in the wild, originating from Hawaii (*G. tomentosum*), Brazil (*G. mustelinum*), and Galapagos (*G. darwinii*), have been widely and extensively used in cotton breeding to improve both yield and fiber quality traits. Wang et al. [12] detected 278 polymorphic loci among 105 ILs by crossing *G. hirsutum* and *G. darwinii*. Wang et al. [13] identified 29 QTLs for fiber quality traits from an advanced backcross population obtained from a cross between *G. hirsutum* and *G. mustelinum*. *G. tomentosum* is a wild tetraploid species that is very closely related to the cultivated allotetraploid species *G. hirsutum* [14]. Zhang et al. [15] exploited the QTL alleles for improved fiber quality traits from *G. tomentosum*, using an advanced backcross population developed from *G. hirsutum* × *G. tomentosum*. Zheng et al. [16] constructed a genetic map using 1295 simple sequence repeat (SSR) markers to locate QTLs for drought-related traits from an interspecific cross between *G. tomentosum* and Upland cotton CRI-12.

The advent of map-based molecular markers and congeneric QTL mapping endorsed the expansion of new molecular approaches to capably exploit the positive alleles present in the wild species [17]. The introgression line libraries were examined by molecular markers, which carry some segments from wild related species in the background among cultivated species, allowing for the representation of entire wild species genomes [18,19]. These lines are obtained by several rounds of continuous backcrossing to its recurrent parent. The development of ILs provides an auspicious opportunity to well utilize the genetic potential of wild species in breeding. These ILs are especially useful for mapping of complex traits because these are permanent populations and could be evaluated in many environments. Additionally, allelic effects of the wild relative donor parent are cultivated in a homogeneous cultivate genetic background that eases the interaction among donor alleles [20,21].

In this study, a mapping population with 107 ILs was developed from the cross between *G. hirsutum acc*. 4105 and *G. tomentosum*. Few reports on cotton introgression breeding using wild species *G. tomentosum* are available for construction of a high-density map [22] and to identify QTLs for drought tolerance [16]. However, all of these studies are usually based on SSR markers. To the best of our knowledge, this is the pioneer study on ILs in cotton breeding using SNP markers. The objectives of the present study were to identify elite lines and to identify favorable QTLs from wild *G. tomentosum* for fiber quality and yield traits. The ILs and QTLs identified in this study can facilitate future molecular breeding programs to improve the fiber quality and yield in Upland cotton.

## 2. Results

### 2.1. Phenotypic Performance of Yield and Fiber Quality Traits in ILs

**Figure 1:**
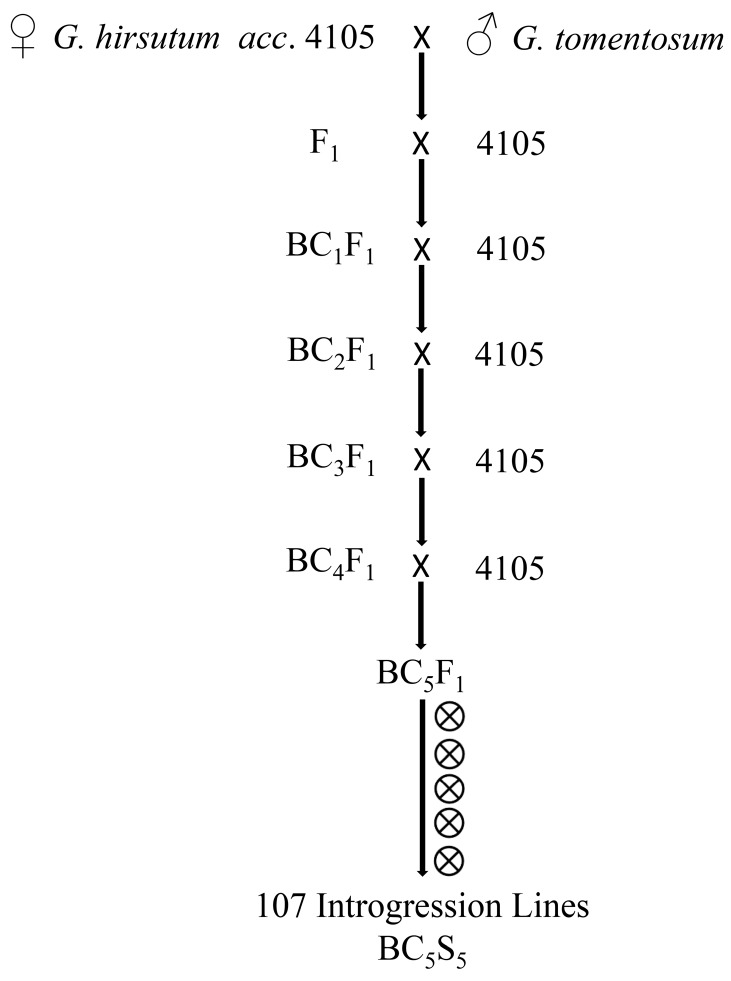
Strategy for development of 107 interspecific hybridization derived ILs used in the study. ⊗ means selfing.

The IL population was developed through interspecific hybridization followed by a series of selfing (Figure 1). The derived IL population with 107 lines with their background parent *G*. *hirsutum acc*. 4105 was tested in three environments for two years. Mean squares revealed significant variations (*p* < 0.01 level) among ILs for all yield and fiber quality traits (Table 1). The variation among the environments was also highly significant for all of the traits, which indicated that the performance of ILs was different across the three environments, while the variation due to genotype × environment was also highly different for all of the traits.

**Figure 2:**
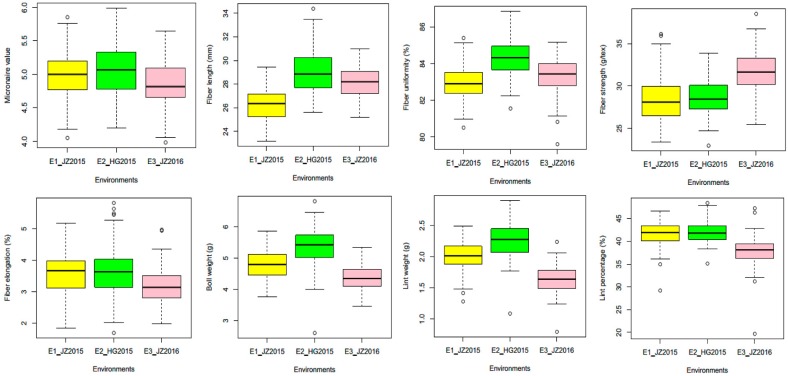
Box plot of the changing trends of fiber quality and yield traits in three environments (HSW, PH, and BN are displayed in the supplementary file; FBN and SI that were only in E3 were not analyzed). Data collected from E1_JZ2015 (ILs planted at Jinzhou in year 2015), E2_HG2015 (at Huanggang in year 2015) and E3_JZ2016 (at Jinzhou in year 2016) are presented, while the circles represent outliers.

The descriptive statistics for the yield and fiber quality traits of the ILs population along with their background parent across three environments are displayed in Table 2. The values of the eight yield and five fiber quality traits showed a large range of variation. The coefficient of variation (CV) values for BN (24.91–25.43) were much higher than those of the other traits, while minimum values (1.27–1.52) of CV were recorded for FU. The results showed that ILs performed more or less equally to their background parent for most of the traits. The skewness and kurtosis were also calculated, and the results revealed that these traits fit a normal distribution. Distribution analysis of the phenotypic values of the thirteen traits showed an incessant normal distribution in the IL population, suggesting that each trait was controlled by multiple genes (Figure S1a,b). The phenotypic trends of eight traits available in three environments are shown in Figure 2; HSW, PH, and BN are shown in Figure S2, (FBN and SI were only in environment 3, thus not analyzed). The variation among these traits across the locations within the same year was less, and the traits were stable, while these traits were influenced by environmental conditions and were less stable across the years. Correlation coefficients among yield and fiber quality traits were calculated and the results are displayed in Table S1. Best linear unbiased predictor (BLUP) values were used to measure the phenotypic correlations among yield and fiber quality traits. The results indicated that FL, FU, and FS exhibited highly significant associations with each other, while MIC showed a significant but negative correlation with FL and it showed negative and non-significant associations with FU and FE. Conversely, FE demonstrated non-significant and negative correlations with MIC and FL, and it had a non-significant relationship with FU and FS. Most of the fiber quality traits showed a non-significant association with the yield traits except MIC, which showed a highly significant association with LP. BW exhibited a highly significant correlation with LW and a significant correlation with HSW and SI. LW was found to have a highly significant relationship with LP and HSW, while HSW demonstrated a significant association with PH and SI. However, PH had significant correlations with BN and FBN.

### 2.2. SLAF-seq and SNP Marker Development

**Figure 3:**
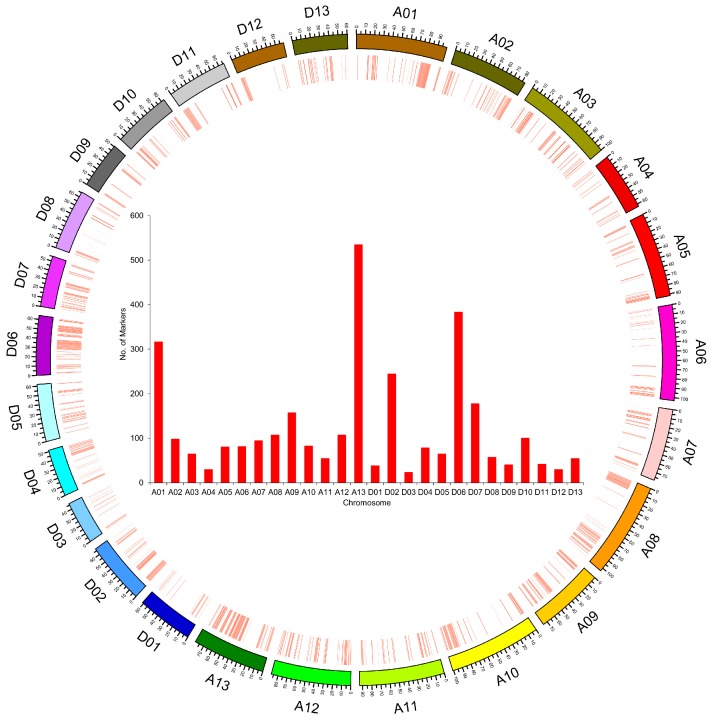
Number of markers in chromosomes. Inside the circle, *x* axis indicates the chromosomes and *y* axis indicates the number of markers. Markers with their positions are assigned to respective chromosomes differentiated by colors.

In total, 265.20 million base (Mb) paired end reads of 100 bp length were obtained in both recurrent and donor parents and 107 ILs. The average Q30 ratio was 91% (average 91% of the bases were of high quality, Q30 means a quality score of 30, indicating a 0.1% chance of an error) and the guanine-cytosine (GC) content was 40% (Table S2). The number of reads in the recurrent parent and donor parent were 19,796,308 and 9,273,354 with a Q30 ratio of 81.87 and 91.1, and the GC content was 41.31 and 40.12, respectively. On average, 2,206,821 reads with a Q30 ratio of 90% and GC content of 37% were generated in ILs. In the recurrent parent, the specific locus amplified fragment (SLAF) number was 595,419, and the average sequencing depth in each marker was 29.73-fold (Figure S3a; Table S3). While for the donor parent, 359,255 SLAFs were obtained with an average depth of 21.12-fold. In the ILs population, the SLAF number was 258,697, and the average depth in each SLAF marker was eight-fold. The average coverage in parents and population revealed that the sequencing results were reliable for marker exploring. Overall, 3157 SNP polymorphic markers were developed and were used for genotyping the whole ILs population and parents. The marker number ranged from 177 to 924 in ILs (Figure S3b). However, SNP markers on each chromosome ranged from 24 to 535 (Figure 3). Chromosome A13 harbor maximum number of markers followed by D06 and A01. Distribution of markers on most of the chromosomes is not uniform, as several larger gaps are shown between the markers. Position of the markers on corresponding chromosome is shown in Figure 3. Maximum SNP markers (1816) were in At, and 1341 markers were in Dt. Statistics for SNP number, hetero-loci, and homo-loci for each ILs is presented in Table S4. The average hetero-loci number in each ILs is 1099.98 with a range of 482–5698, while average homo-loci number in each ILs is 13,583.04 with a range of 9329–14,948. However, hetero-loci ration in each ILs ranged from 0.035 to 0.379 with an average of 0.073, and average homo-loci ratio in each ILs is 0.926, ranging from 0.620 to 0.964.

### 2.3. Genomic Component and Diversity of the ILs

**Figure 4:**
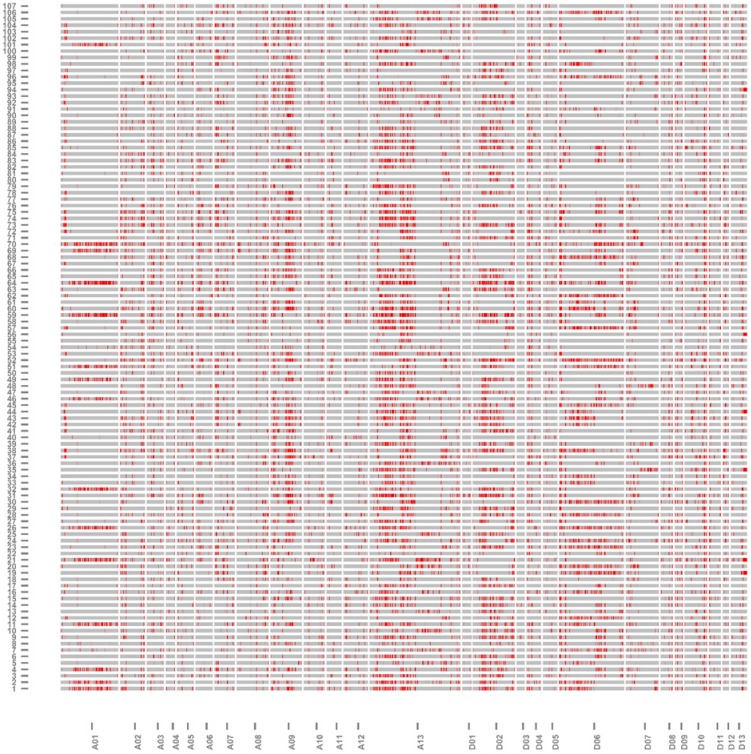
Graphic of genotypes of the 107 ILs. Red regions represent introgression segments from *G. tomentosum.*

**Figure 5:**
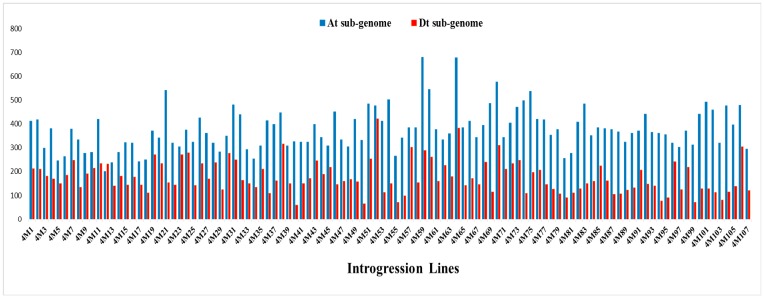
Introgression length in the individual IL population in the At and Dt.

The physical distance, coverage of the introgressed segments in the genome and percentage of genome coverage were calculated by using Microsoft Excel. The average physical distance in the At is 88.48 Mb, ranging from 62.91 to 103.63 Mb (Table 3). However, the average physical distance in the Dt is 59.57 Mb, ranging from 46.69 to 67.28 Mb. The introgressed segments represented 35.13% of the genome of tetraploid cotton, with 35.68% and 34.59% in the At and Dt, respectively. The highest coverage of introgressed segments was 62 Mb on chromosome A13, while the lowest was 9 Mb on chromosome D09. The maximum percentage of genome coverage was on A13 (77.54%), and the minimum percentage of coverage was on D11 (19.67%) (Figure 3 and Table 3). The introgression segments from wild parent in the each IL and on each chromosome are shown in Figure 4. The introgressed components varied in length among the ILs (Figure 5). The average introgressed length in the At in the ILs was 376.22 Mb and ranged from 201 to 679 Mb. The maximum introgression was in the introgression line 4M59, while the minimum introgression length was in the introgression line 4M12. However, the average introgression length in the ILs in the Dt was 176.64 Mb, ranging from 60 to 422 Mb. The highest introgression was in line 4M52, and the lowest introgression was in line 4M40.

### 2.4. QTL Mapping for Fiber Quality and Yield Traits

A total of 74 QTLs for fiber quality and yield were detected on 20 chromosomes. Of these, 30 QTLs were detected for five fiber quality traits, and 44 QTLs for eight yield traits. From the 74 QTLs, 47 QTLs (63.51%) were identified in the At, and 27 QTLs (36.49%) were in the Dt. Overall, 69 SNP markers were found to be associated with 74 QTLs. Twenty-nine markers were associated with fiber quality traits, and forty markers were associated with yield. The logarithm of odds (LOD), position, percentages of PVE, and additive effects of QTLs are presented in Table S5. On average, the PVE in 13 traits was 10.22%, ranging from 2.02% to 30.15%.

There were four QTLs for MIC on four chromosomes (A05, A06, D03, and D11), with the LOD scores ranging from 3.02 to 34.00. The additive effects of two QTLs (*qMIC-A06*-1 and *qMIC-D11-1*) increased the micronaire with PVE values of 10.04% and 12.17%, while the remaining two QTLs (*qMIC-A05-1* and *qMIC-D03-1*) had decreasing additive effects on micronaire with PVE values of 8.92% and 13.57%.

For FL, 10 QTL effects were detected on seven chromosomes (A03, A10, A12, A13, D05, D06, and D10) with PVE ranging from 2.73% to 14.59%. Four QTLs (*qFL-A03-1*, *qFL-A10-1*, *qFL-A12-1*, and *qFL-D06-1*) showed increasing effects for fiber length, and six QTLs (*qFL-A12-2*, *qFL-A13-1*, *qFL-D05-1*, *qFL-D06-2*, *qFL-D10-1*, and *qFL-D10-2*) had decreasing effects. Three chromosomes (A12, D06, and D10) each harbored two QTLs, while four chromosomes (A03, A10, A13, and D05) each harbored one QTL.

Only one QTL for FU (*qFU-A06-1*) was identified on chromosome A06, with decreasing effects on fiber uniformity. The PVE of this QTL was 17.94%.

A total of six QTLs for FS were detected on three chromosomes (A01, A09, and D02). A01 harbored three QTLs, A09 harbored two QTLs and D02 harbored only one QTL. PVE ranged from 8.45 to 21.61%, and four QTLs (*qFS-D02-1*, *qFS-A01-1*, *qFS-A09-1*, and *qFS-A09-2*) among them had increasing effects, while the remaining two QTLs (*qFS-A01-1* and *qFS-A01-3*) decreased the FS.

In total, nine QTLs were detected for FE on eight chromosomes (A02, A11, A12, A13, D02, D06, D07, and D08), with PVE ranging from 8.78% to 15.12% for each QTL. Eight QTLs (*qFE-A08-1*, *qFE-A11-1*, *qFE-A12-1*, *qFE-D06-1*, *qFE-A02-1*, *qFE-D02-1*, *qFE-D02-2*, and *qFE-D07-1*) had positive additive effects, and only one QTL (*qFE-A13-1*) was identified with negative additive effects.

There were seven QTLs detected for BW on six chromosomes (A03, A07, A09, A13, D04, and D10), with the LOD scores ranging from 3.05 to 24.26. The average PVE value for these seven QTLs was 11.97, ranging from 8.25 to 19.53. Chromosome A09 harbored two QTLs. The additive effects of three QTLs (*qBW-D04-1*, *qBW-A09-1*, and *qBW-A09-2*) increased the BW, with PVE values of 13.33%, 8.44%, and 11.42%, respectively, while the remaining four QTLs (*qBW-A07-1*, *qBW-A10-1*, *qBW-A03-1*, and *qBWC-A13-1*) had decreasing additive effects, with PVE values of 19.53%, 8.25%, 10.97%, and 11.83%, respectively (Table 3).

For LW, three QTLs were detected on three chromosomes (A05, A07, and D10) with PVE ranging from 10.46% to 16.41%. The LOD values of these QTLs ranged from 3.43 to 5.15. All three QTLs (*qLW-A07-1*, *qLW-D10-1*, and *qLW-A05-1*) had decreasing effects.

In total, 13 QTLs were detected for LP on nine chromosomes (A01, A05, A07, A11, A12, A13, D02, D05, and D06). Chromosomes A07, A11, and A13 each harbored two QTLs, and the remainder harbored only a single QTL. The PVE values ranged from 2.02% to 30.15% for each QTL. LOD scores ranged from 3.70 to 29.58 for these QTLs. Seven QTLs (*qLP-A01-2*, *qLP-A07-2*, *qLP-A11-1*, *qLP-A12-1*, *qLP-A13-1*, *qLP-D02-1*, and *qLP-D05-1*) had positive additive effects, and six QTLs (*qLP-A07-1*, *qLP-A01-1*, *qLP-A05-1*, *qLP-A11-2*, *qLP-A13-2*, and *qLP-D06-1*) were identified with negative additive effects.

In total, 11 QTLs for HSW were detected on eight chromosomes (A02, A03, A06, A09, A13, D02, D06, and D07). Chromosome A13 harbored three QTLs, D06 harbored two QTLs, and the remaining chromosomes harbored only one QTL. LOD scores for these QTLs ranged from 3.15 to 15.09, and PVE ranged from 2.82% to 17.77%. Eight QTLs (*qHSW-A02-1*, *qHSW-A03-1*, *qHSW-A06-1*, *qHSW-A09-1*, *qHSW-A13-2*, *qHSW-A13-3*, *qHSW-D06-2*, and *qHSW-D07-1*) had increasing effects, while the remaining three QTLs (*qHSW-A13-1*, *qHSW-D02-1*, and *qHSW-D06-1*) decreased the HSW.

Three QTLs for PH on three chromosomes (A13, D06, and D11) were detected with PVE ranging from 10.93% to 16.08% for each QTL. LOD scores ranged from 3.13 to 4.47. Two QTLs (*qPH-D11-1* and *qPH-A13-1*) had positive additive effects, and *qPH-D06-1* was identified with negative additive effect.

There were four QTLs for BN on three chromosomes (A01, A13, and D02), with PVE ranging from 1.74% to 15.61% for each QTL. LOD scores ranged from 3.26 to 6.27. Chromosome A13 harbored two QTLs, and the remaining chromosomes harbored a single QTL each. Three QTLs (*qBN-A01-1*, *qBN-A13-2*, and *qBN-D02-1*) had positive additive effects, and only one QTL (*qBN-A13-1*) was identified with negative additive effect.

Only one QTL for FBN (*qFBN-D02-1*) was identified on chromosome D02 with decreasing effects. The PVE of this QTL was 13.07%, and the LOD score was 3.02.

Two QTLs for SI on two chromosomes (A02 and A13) were detected with PVE values of 10.83 and 18.10% for each QTL. LOD scores of these QTLs were 3.62 and 6.17, respectively. Both QTLs (*qSI-A02-1* and *qSI-A13-1*) had positive additive effects.

### 2.5. QTL Hotspot Analysis

For identification of significant genomic regions that harbor multiple QTLs associated with the important fiber quality and yield traits, the positions of all of the QTLs were specified on the chromosomes. Said et al. [23] conducted meta QTL analysis and suggested that a QTL cluster can be assigned to 20 cM region amid presence of more than two stable QTLs. Moreover, integration of interspecific genetic map [24] estimated that on average 1cM equated ~0.5 Mb physical region on cotton genome. Here we considered 10 Mb (~20 cM) physical region enclosing two or more QTLs as a cluster. Five QTL clusters on five chromosomes were detected with at least three QTLs in each cluster (Table 4). Three clusters were in the At, and two were in the Dt. All of these clusters contained QTLs for more than one different fiber quality or yield trait. The highest number of QTLs was five in A01-cluster and A13-cluster, each in the At for FS, LP, BN, HSW, PH, and SI. These QTLs with increasing effects could simultaneously improve the yield with acceptable fiber quality in Upland cotton.

## 3. Discussion

### 3.1. Advantage of the Permanent Population Derived from Wild Species

Simultaneous improvement of cotton yield with good fiber quality is highly desired in cotton breeding. Nonetheless, successes in such breeding programs are restricted by the absence of promising alleles considering outstanding fiber quality and yield in gene pool of Upland cotton [25]. Use of introgression lines with better yield potential and superior fiber quality is one of the key strategies for improving Upland cotton in terms of both higher yield and good fiber quality. Although wild species are morphologically inferior, they possess superior alleles which have been gone behind in domestication and are frequently discovered by the poor genetic background. The fiber quality potential of elite cotton varieties can further be improved when these superior alleles are transferred into elite cotton cultivars. Thus, IL population offers an opportunity for efficient utilization of the genetic potential of the wild species. Zamir [18] described that the complete set of ILs was supposed to exemplify the whole exotic genome, while each single line possessed chromosome segments from the exotic parental line and rest of the genome was consistently obtained from an elite variety.

In present research, an ILs population with 107 lines was developed with five rounds of backcrossing, each hypothetically minimizing exotic genomic portion by 50% in the following generations. Five selfings have been successively executed to obtain complete homozygous lines that are the stable genetic resource for further evaluation. A wild allotetraploid species *G. tomentosum* was used as donor parent, and the main reason behind selection of *G. tomentosum* as male parent was to utilize the desirable exotic genes for the improvement of Upland cotton. In this study, the ILs population is a rich genetic material which possesses great diversity with introgressed segments from wild *G. tomentosum* species, and introgressed segments represent 34.54% of the genome of tetraploid cotton. The coverage of introgressed segments is much higher in the At than in the Dt. The ILs population showed a large range of variability for the fiber quality and yield traits. Introgression segments from *G. tomentosum* have greatly affected both fiber quality and yield in ILs. More than half of the ILs were found to be with better fiber quality traits and improved yield. This IL population was used to dissect genetic architecture of the fiber quality and yield traits in this study, and this population will serve as vital genomic resource for cotton breeding and QTL fine mapping.

### 3.2. Characteristics of SLAF-Sequencing Strategy in Genotyping ILs

Previous studies in cotton using ILs were usually based on SSR markers. Numerous studies about QTL mapping for the improvement of fiber quality and yield traits in the *Gossypium* genus using SSRs have been reported using germplasm having introgressed genomic segments from their wild relative species [12,26]. For high resolution of QTL mapping, traditional markers such as SSRs are not sufficient when their linkage distance is zero, and thus, they cannot meet the requirements [27].

Polyploid crops have large genomes along with large-scale repetitive sequences; as a result, there are many challenges in developing SNPs for polyploidy crops. With the fast progress of whole genome sequencing technologies, sequencing-based marker discovery and genotyping technologies make it possible and have provided good opportunities to develop high-throughput and large-scale SNP markers in many genetic studies [28]. SNPs are ample and are a very stable type of genetic variation, which are characterized as having lower mutation rates, higher numbers, and higher accuracy [29,30]. This has led to the discovery of superior high-density SNP gene-chip technology, which is developed as a superior method for linkage mapping and QTL detection. Now, it is being used extensively to detect QTLs in bi-parental populations of many crop species [31].

SLAF sequencing is a recently developed high-resolution technique to obtain SNPs in large numbers and to perform genotyping by high-throughput sequencing [32]; it combines both high-throughput sequencing and specific locus amplification, and it has been successively and widely used in several plant species [32,33,34,35]. The results obtained from these studies have proven that SLAF-seq is a strong high-throughput method to develop large numbers of SNP markers in a limited time. Compared to other methods of SNP marker development, SLAF-sequencing technology has many significant advantages, such as uniformity, high accuracy, stability, high rate of success, and low cost. Additionally, it does not necessarily require full genomes sequences or genome SNPs [32]. In cotton, few reports of SLAF-seq are available regarding the construction of high-density genetic maps [36], identification of favorable alleles and candidate genes [37], or comprehensive analysis of polymorphisms among tetraploid cotton species [38]. In this study, a SLAF-seq strategy was used in the ILs population in order to identify QTLs for fiber quality and yield from wild relative species. Taking advantage of massively parallel sequencing technology, 265.20 Mb pair-end reads were generated by the SLAF method. After the pre-design scheme, a pilot experiment for ensuring the marker density, uniformity, and efficiency, and filtering low-depth SLAF tags was performed, and eventually, 3157 polymorphic SNP markers were identified. The integrity and the precision of SLAF markers were higher. The distribution of markers was uneven, as more markers (1816) were identified in the At than in the Dt (1341). Few chromosomes, such as A13 followed by D06 and A01, harbored large numbers of markers, which might be due to the large number of introgressed segments on these chromosomes. In initial steps of marker development using SLAF-sequencing, SLAF quantities were more or less same sizes in the chromosomes. After several steps of screening and stringent criteria of SNP filtration, the number of SNP markers varied greatly, and SNP marker ratio reduced on some chromosomes, which led to uneven distribution of markers among the chromosomes. The unbalanced chromosomal dissemination of marker loci is possibly the result of the larger genome size of the At. The results obtained in this study clearly demonstrated that SLAF sequencing is a suitable tool for rapid development of efficient markers in large numbers and large-scale genotyping.

### 3.3. QTL Mapping Using SNP Markers in ILs

Fiber quality and yield traits are controlled by polygenes, and the QTLs identified tend to vary in diverse environments. For identification of more stable and convincing QTLs in multiple environments, permanent populations such as ILs are needed. The ILs are an ideal population for QTL mapping of the complex traits because they possess the potential to reveal new alleles from the wild landraces, to identify genes, and to develop genome-wide genetic resources [39]. It is sensible that the higher yield and superior fiber quality traits of ILs are associated with introgressed genomic components or separated introgression alleles. Therefore, identifying the QTLs for both yield- and fiber quality-related traits associated with introgressed genomic components by molecular markers could provide a better understanding of the genetic mechanism of the introgressed segments on yield and fiber quality traits.

In this study, 74 QTLs were detected using ICIM software 4.1 (http://www.isbreeding.net). Of these, 30 QTLs were detected for five fiber quality traits, and 44 QTLs were detected for eight yield traits. The QTLs were not distributed uniformly in the At and Dt. Of the 74 QTLs, 47 QTLs (63.51%) were identified in the At compared with the Dt (27 QTLs, 36.49%). Previous studies using meta-analysis [5,40,41] have reported that in cotton, a higher number of QTLs for fiber traits exist in the Dt chromosomes. Yu et al. [7] also observed 35% more QTLs in the Dt in an interspecific backcross inbreed line (BIL) population. However, our results are inconsistent with these previous reports. This observation might possibly be due to the use of different populations and different markers, and genes related to adaptation or domestication should be more common in the At because these ILs need to produce more seed for survival. Two QTLs (*qFS-A09* and *qLP-A07*) were detected in two environments. Significant environmental effects were observed for fiber quality and yield traits and different climatic conditions have been observed to affect the fiber quality and yield of cotton. This might be the reason why we were unable to detect more stable QTLs in this study. In this study, PVE in 13 different traits was relatively low (10.22%), ranging from 2.02% to 30.15%. Low PVE (3.18%) ranging from 1.05% to 12.67% for six different yield and fiber traits using introgression lines was also reported by Si et al. [42]. More than half, 44 QTLs (59.46%), in this study showed positive additive effects for fiber quality and yield traits, suggesting that segments from the donor parent improved both the fiber quality and yield significantly, and these ILs could be used for improvement of the desired yield and fiber quality in *G. hirsutum* cultivars. Because this is a pioneer study in employing high-throughput sequencing by SLAF in cotton using ILs population, the results obtained are unique compared to previous reports, and it is difficult to compare our results with previous results.

Co-localization of QTL on chromosomes, referred to as QTL clusters, were detected for fiber quality and yield traits in this study, indicating that the pleiotropic loci may control these traits. QTL co-localization on chromosomes, referred to as “QTL cluster/hotspots”, have previously reported in cotton [5,42]. In the present research, a few genomic regions containing QTL clusters were examined, mainly on chromosomes A01, A09, A13, D02, and D10. These QTL clusters affected two or more different fiber quality and yield traits. The highest number of QTLs was observed on A01-cluster and A13-cluster, where five QTLs were detected on each cluster for FS, LP, BN, HSW, PH, and SI. These co-localized QTLs might elucidate the phenotypic correlation measured. These QTL clusters have provided some valuable information to define genome regions with different traits. Based on the comprehensive analysis of clusters in this study, breeding programs targeting higher yields with superior fiber quality can focus on hotspot clustering areas and select around the region. The existence of hotspots and QTL clusters has proven that genes related to certain traits were more heavily focused in certain areas of the genome than others [5,43].

## 4. Materials and Methods

### 4.1. Plant Materials

In this study, an interspecific BC_5_S_5_ introgression population with 107 lines (designated as 4M ILs) was developed. *G. hirsutum acc*. 4105, a high-yield Upland cotton line, was used as the recurrent parent, while *G. tomentosum* was used as the donor parent. The F_1_ plants were crossed with its recurrent parent as the female parent to produce BC_1_F_1_ individuals. The BC_5_F_1_ population was developed with a series of backcrosses to its recurrent parent as the female parent. Thereafter, BC_5_F_1_ individuals were continuously self-fertilized to produce the BC_5_S_5_ introgression population (Figure S3). All of the ILs along with the background parent were planted in Huanggang and Jinzhou, Hubei province, in 2015, and in Jinzhou and Ezhou (crop was destroyed by flood and waterlogging in Ezhou), Hubei province, in 2016. A randomized complete block design with two replications was applied in each environment. Ten plants were maintained in one row for each line per replication. The essential cultural operations were adopted uniformly in all of the plots throughout the growing period. All agronomic practices such as standard cultivation, weed and insect control practices were followed at the proper time throughout the growing season.

### 4.2. Phenotypic Traits Collection and Analysis

The phenotypic performance of ILs was assessed in eight yield and five fiber quality traits across the three environments. Twenty bolls at maturity from middle fruiting branches of the plants from each line per replication in each environment were collected, and yield traits were determined by weighing the samples and adopting equations. The yield traits included boll weight (BW), lint weight (LW), lint percentage (LP), 100-seed weight (HSW), plant height (PH), number of bolls plant^−1^ (BN), number of fruiting branches (FBN), and seed index (SI). Data for HSW, PH, and BN were missing in E1, and FBN and SI were only available in E3 and analyzed accordingly. For the measurement of fiber quality traits, 10–15 g fiber samples after ginning from every line along with their parental line were sent to the Institute of Cotton Research, Shihezi Academy of Agricultural Sciences, Xinjiang, for testing fiber quality. The fiber quality traits were tested with an HVI-1000 Automatic Fiber Determination System (USTER^®^ HVI 1000, Uster Technologies, Uster, Switzerland) at 20 °C and at relative humidity of approximately 65. Traits included the micronaire value (MIC), fiber length (FL), fiber uniformity (FU), fiber strength (FS), and fiber elongation (FE). The analysis of variance and basic statistics such as the mean squares, standard deviation, standard error, skewness, and kurtosis analysis for yield and fiber quality traits were calculated using the STATISTIX 8.1 package (Analytical Software 2005, v8.1, Tallahassee, FL, USA). In addition, correlation coefficients (*r*) among yield and fiber quality traits were also calculated. The frequency distribution of traits was analyzed by using SPSS version 20.0 (SPSS, Chicago, IL, USA), and the analysis of phenotypic changing trends and the relevance of yield and fiber quality traits were shown in a box plot produced by using the “R” program.

### 4.3. DNA Extraction, SLAF-Library Construction, and High throughput Sequencing

The young and healthy fresh leaf samples from *G. hirsutum acc*. 4105 and each IL were collected and kept at −70 °C. The total genomic DNA was extracted by using a TIANGEN Plant Genomic Kit (TIANGEN Biotech, Beijing, China). The concentration of DNA was tested with a NanoDrop-2000 Spectrophotometer (NanoDrop, Wilmington, DE, USA), and the quality of DNA was also determined by the agarose gel electrophoresis (1%). The procedure for construction of the SLAF library was executed according to Shen et al. [38]. Initially, a pilot experiment was performed for evaluation of the enzymes and to determine the sizes of the restriction fragments for preparing maximum quantity and high quality SLAFs. For SLAF sequencing, four criteria were selected: (i) the SLAF number must be as low as possible in the repeated regions; (ii) SLAFs must be consistently disseminated in the whole genome; (iii) the SLAF length should be appropriate for an exact experimental system; and (iv) the final SLAF number must meet to the expectations. Then, based on the results obtained from the pilot experiment, the SLAF library was constructed. A cotton reference genome [8] was used. The clean DNA was digested into fragments with a size of 314–344 bp with the specific enzyme combinations Hae+Rsal (NEB, Ipswich, MA, USA.). Afterwards, fragments end amends, indexed paired-end adapters, ligation, and adjusted ends obtainment were then performed step by step accordingly. Objective size was selected on a 2% agarose gel and subjected to amplification of the fragments through reaction. Finally, high-throughput sequencing was performed using an Illumina HiSeqTM-2500 (Illumina, Inc., SanDiego, CA, USA) at the Biomarker Technologies Corporation in Beijing. A real-time examination was performed and the ratio of the high quality with quality scores higher than the Q30 (indicating the chance of an error of approximately 0.1% that means 99.9% of the confidence) in the raw reads was calculated, while guanine-cytosine (GC) amounts for the quality control was measured. The SLAF-sequences of *G. tomentosum* were from Shen et al. [38].

### 4.4. SLAF-seq Data Grouping and Genotyping

The identification of the SLAF markers and genotyping was done according to the procedures described by Shen et al. [38]. In brief, raw reads were arranged for the progenies according to the duplex barcode sequences, and low-quality reads (quality score ˂ 20 e) were first filtered out. Then, each of the high-quality reads was trimmed off by 5 bp at the terminal site [36]. The reference genome TM-1 sequence was downloaded from the CottonGen database (https://www.cottongen.org). Clean reads from each sample were achieved and mapped to the *G. hirsutum* TM-1 genome [8] using Burrows-Wheeler-Aligner (BWA, v0.7.10) software [44]. The mapped reads with high mapping quality (MQ ≥ 20) and high base quality (Q ≥ 30) were considered for downstream analysis [38]. Genome Analysis Toolkit (GATK) software [45], Samtools/bcftools [46,47] were used to detect SNPs with default parameters. Furthermore, in order to minimize detection of the false positives when calling SNPs, the stringent parameters of the software were used. SNPs were filtered with the criteria that the minimum read depth was less than 10, and the average base quality was less than 30 [38]. Between two parents, 25,659 SNPs were identified, and 20,370 SNPs were on the chromosome. Then, the SNPs in each ILs having the different position between the parents were further filtered. Finally, only 3157 SNPs were obtained on the chromosome with consistency in the parents. CIRCOS 0.66 software was used to estimate the positions of markers on physical map [48]. The genome sequences of ILs developed from SLAF-seq are available at NCBI Sequence Read Archive database with BioProject accession number PRJNA421265 and PRJNA316549 [38].

### 4.5. QTL Analysis for Yield and Fiber Quality Traits

A likelihood test based on stepwise regression (RSTER-LRT) was used to identify QTLs in ILs [49]. The software QTL IciMapping 4.1 (http://www.isbreeding.net) was used to detect QTL effects. An LOD threshold of 3.0 was considered to define significant additive QTLs. QTLs were named as follows: q + trait abbreviation + chromosome number + QTL number [50].

## 5. Conclusions

In conclusion, we used a population of 107 ILs and confirmed the potential of this population for QTL identification of fiber quality and yield traits. Seventy-four QTLs related to different fiber quality and yield traits were identified. Five QTL clusters were identified that could be used in further breeding programs for improving the fiber quality with high yield in the Upland cotton. This IL population can be used for the mapping and cloning of the novel QTL/genes that control corresponding desired traits and will serve as a rich source of plant materials for the cotton research community.

## Figures and Tables

**Table 1:** Mean squares of various fiber quality and yield traits revealed by Analysis of Variance (ANOVA).

**Source of Variation**	**Replication (R)**	**ILs**	**Environment (E)**	**ILs × E**	**Error**
	**D.F. = 1**	**D.F. = 106**	**D.F. = 2**	**D.F. = 214**	**D.F. = 321**
MIC	0.147	0.266 **	2.361 **	0.242 **	0.083
FL	0.054	3.948 **	451.793 **	3.003 **	1.141
FU	0.379	1.530 **	101.344 **	1.534 **	1.066
FS	14.395	12.410 **	650.072 **	10.213 **	5.096
FE	0.284	0.953 **	14.661 **	0.767 **	0.341
BW	0.812	0.410 **	47.524 **	0.321 **	0.174
LW	0.021	0.112 **	19.035 **	0.075 **	0.010
LP	54.740	15.260 **	1216.613 **	13.021 **	9.182
**Source of Variation**	**Replication (R)**	**ILs**	**Environment (E)**	**ILs × E**	**Error**
	**D.F. = 1**	**D.F. = 106**	**D.F. = 1**	**D.F. = 106**	**D.F. = 213**
HSW	6.481	1.382 **	5243.981 **	1.679 **	0.921
PH	108.001	229.215 **	317,162.146 **	167.541 **	109.551
BN	444.164	123.641 **	17,213.992 **	121.418 **	92.814
**Source of Variation**	**Replication (R)**	**ILs**			**Error**
	**D.F. = 1**	**D.F. = 106**			**D.F. = 105**
FBN	86.580	76.332 **			29.093
SI	1.616	1.169 **			0.482

**: Significant at *p* < 0.01, MIC: micronaire value, FL: fiber length, FU: fiber uniformity, FS: fiber strength, FE: fiber elongation, BW: boll weight, LW: lint weight, LP: lint percentage, HSW: hundred seed weight, PH: plant height, BN: boll number, FBN: fruiting branches number, SI: seed index. D.F.: degrees of freedom.

**Table 2:** Descriptive statistics of values for various fiber quality and yield traits across three environments.

Trait	Env.	4105 Mean	ILs Mean	SD	CV (%)	Skewness	Kurtosis
MIC	E1	5.07	4.99	0.38	8.35	−0.20	−0.37
	E2	-	5.08	0.43	8.60	−0.16	−0.30
	E3	4.81	4.85	0.33	7.80	0.04	0.16
FL	E1	26.99	26.29	1.26	5.34	0.25	−0.48
	E2	-	29.09	1.52	5.53	0.39	0.30
	E3	28.23	28.23	1.29	5.21	0.26	−0.31
FU	E1	84.21	82.96	0.86	1.27	−0.01	−0.04
	E2	-	84.31	0.91	1.28	−0.15	0.11
	E3	83.69	83.31	0.95	1.52	−0.60	0.65
FS	E1	30.46	28.42	2.76	11.11	0.50	0.53
	E2	-	28.77	2.16	8.51	0.35	0.01
	E3	30.86	31.76	2.39	9.14	−0.06	0.44
FE	E1	3.18	3.63	0.72	21.72	0.17	0.05
	E2	-	3.66	0.78	23.29	0.35	0.45
	E3	2.82	3.20	0.56	21.57	0.29	0.48
BW	E1	4.47	4.81	0.49	10.39	0.12	−0.04
	E2	-	5.31	0.58	10.93	−0.83	2.70
	E3	4.05	4.36	0.45	10.48	0.22	1.77
LW	E1	1.85	2.01	0.25	12.43	−0.29	0.20
	E2	-	2.23	0.26	11.95	−0.63	1.17
	E3	1.5	1.64	0.24	14.66	1.14	0.68
LP	E1	42.82	41.80	2.76	6.60	−1.01	1.97
	E2	-	42.02	2.70	6.42	0.82	2.93
	E3	37.08	37.82	4.48	11.86	-1.39	−1.56
HSW	E2	10.85	9.97	0.81	8.18	0.055	0.39
	E3	17.2	16.93	1.35	8.00	0.07	−0.53
PH	E2	142.20	139.68	13.99	10.021	0.06	−0.48
	E3	80	84.82	10.24	12.08	0.43	0.73
BN	E2	39.25	35.11	8.93	25.43	0.98	1.61
	E3	59.5	47.36	11.79	24.91	0.42	0.33
FBN	E2	19.71	17.44	2.60	14.92	−1.28	0.21
SI	E3	10.6	10.44	0.90	8.70	0.11	−0.05

E1, Jinzhou2015; E2, Huanggang2015; E3, Jinzhou2016; Env., environment; ILs, Introgression lines; SD, Standard deviation; CV, Coefficient of variation.

**Table 3:** Genome coverage of introgressed chromosome segments in ILs.

Chromosome	Size of Physical Distance (Mb)	Coverage of Introgressed Segments in Genome (Mb)	Percentage of Genome Coverage (%)
A1	99.88	38	38.04
A2	83.45	34	40.74
A3	100.26	22	21.94
A4	62.91	13	20.66
A5	82.05	29	35.35
A6	103.17	24	23.26
A7	78.25	24	30.67
A8	103.63	39	37.64
A9	75.00	30	40.00
A10	100.87	29	28.75
A11	93.32	19	20.36
A12	87.48	40	45.72
A13	79.96	62	77.54
Average	88.48	31.00	35.44
D1	61.46	15	24.41
D2	67.28	34	50.53
D3	46.69	9	19.28
D4	51.45	22	42.76
D5	61.93	21	33.91
D6	64.29	38	59.10
D7	55.31	32	57.85
D8	65.89	14	21.25
D9	51.00	12	23.53
D10	63.37	19	29.98
D11	66.09	13	19.67
D12	59.11	12	20.30
D13	60.53	21	34.69
Average	59.57	20.15	33.64

**Table 4:** Distribution of quantitative trait loci (QTL) clusters on the chromosomes for fiber quality and yield traits.

Cluster Name	Approximate Position on Chromosome (Mb)	Number of QTLs	Name of QTLs
A01-cluster	77.52–86.96	5	*qFS-A01-1*
			*qFS-A01-2*
			*qLP-A01-1*
			*qLP-A01-2*
			*qBN-A01-1*
A09-cluster	45.81–46.41	3	*qFS-A09-1*
			*qFS-A09-2*
			*qBW-A09-1*
A13-cluster	48.09–58.85	5	*qLP-A13-1*
			*qHSW-A13-1*
			*qHSW-A13-2*
			*qPH-A13-1*
			*qSI-A13-1*
D02-cluster	18.22–22.44	4	*qFS-D02-1*
			*qFE-D02-1*
			*qLP-D02-1*
			*qFBN-D02-1*
D10-cluster	56.48–61.85	3	*qFL-D10-2*
			*qBW-D10-1*
			*qLW-D10-1*

## Supplementary Materials

Supplementary materials can be found at www.mdpi.com/1422-0067/19/1/243/s1.

## Abbreviations

ILs	Introgression lines
SLAF	Specific locus amplified fragment
SNP	Single nucleotide polymorphism
QTL	Quantitative trait loci
At	A sub-genome
Dt	D sub-genome
PVE	Phenotypic variance explained
MIC	Micronaire value
FL	Fiber length
FU	Fiber uniformity
FS	Fiber strength
FE	Fiber elongation
BW	Boll weight
LP	Lint percentage
HSW	Hundred seed weight
PH	Plant height
BN	Boll number
FBN	Fruiting branches number
SI	Seed index
CV	Coefficient of variance
E1-3	Environment 1-3
LOD	Logarithm of odds
