# Supplementary material for: QTL Mapping for Fiber Quality and Yield Traits Based on Introgression Lines Derived from Gossypium hirsutum × G. tomentosum
Source: Int J Mol Sci. 2018 Jan 14;19(1):243. doi: 10.3390/ijms19010243 (PMC5796191; doi:10.3390/ijms19010243)
Supplement: Supplementary file 1 [file ijms-19-00243-s001.pdf]

Supplementary Materials: QTL mapping for fiber quality and yield traits based on introgression lines derived from *Gossypium hirsutum* × *G. tomentosum*

Ayaz Ali Keerio, Chao Shen, Yichun Nie, Muhammad Mahmood Ahmed, Xianlong Zhang and Zhongxu Lin

Table S1. Correlation coefficient among fiber quality and yield traits.

Traits	MIC	FL	FU	FS	FE	BW	LW	LP	HSW	PH	BN	FBN
FL	−0.41 **	-	-	-	-	-	-	-	-	-	-	-
FU	−0.04 NS	0.50 **	-	-	-	-	-	-	-	-	-	-
FS	0.01 NS	0.58 **	0.62 **	-	-	-	-	-	-	-	-	-
FE	−0.01 NS	−0.09 NS	0.04 NS	0.14 NS	-	-	-	-	-	-	-	-
BW	−0.06 NS	0.02 NS	−0.02 NS	−0.07 NS	−0.21 *	-	-	-	-	-	-	-
LW	0.16 NS	−0.17 NS	−0.07 NS	−0.16 NS	−0.15 NS	0.83 **	-	-	-	-	-	-
LP	0.40 **	−0.35 *	−0.10 NS	−0.21 *	0.01 NS	0.08NS	0.61 **	-	-	-	-	-
HSW	0.06 NS	−0.14 NS	0.01 NS	0.01 NS	−0.07 NS	0.20 *	0.25 *	0.07 NS	-	-	-	-
PH	0.06 NS	−0.07 NS	0.03 NS	−0.05 NS	−0.02 NS	0.05 NS	0.10 NS	0.11 NS	0.23 *	-	-	-
BN	0.12 NS	0.09 NS	0.13 NS	0.02 NS	0.01 NS	−0.21 NS	−0.10 NS	0.07 NS	0.03 NS	0.28 *	-	-
FBN	0.05 NS	0.09 NS	0.03 NS	0.04 NS	−0.04 NS	−0.10 NS	−0.13 NS	−0.09 NS	0.07 NS	0.29 *	0.18 NS	-
SI	−0.17 NS	0.07 NS	0.09 NS	−0.04 NS	−0.04 NS	0.26 *	0.16 NS	−0.10 NS	0.41 **	0.09 NS	−0.02 NS	−0.04 NS

** = Significant at $p < 0.01$; * = Significant at $p < 0.05$; NS = Non-significant.

Table S2. Basic characteristics of SLAF library construction.

Samples	Total Reads	Q30 Percentage (%)	GC Percentage (%)
4105 (Background parent)	19,796,308	91.87	41.31
G. tomentosum	9,273,354	91.1	40.12
ILs	2,206,821	90	37
Average	-	91	40

Data shown for ILs are average of all the lines.

Table S3. Distribution of SLAF number and sequencing depth in parents and ILs.

Samples	SLAF Numbers	Total Depth	Average Depth
4105 (Background parent)	595,419	17,700,686	29.73
G. tomentosum	359,255	7,587,937	21.12
ILs	258,697	2,030,437	8

Data shown for ILs are average of all the lines.

Table S4. Statistics on the number of SNP for each ILs

ILs	SNP Number	Heter-Loci Number	Homo-Loci Number	Heter-loci Ratio	Homo-loci Ratio
1	14,647	582	14,065	3.97%	96.03%
2	14,583	900	13,683	6.17%	93.83%
3	15,006	707	14,299	4.71%	95.29%
4	13,989	632	13,357	4.51%	95.48%
5	14,297	1334	12,963	9.33%	90.67%
6	15,067	630	14,437	4.18%	95.82%
7	15,027	5698	93,29	37.91%	62.08%
8	13,998	1961	12,037	14.00%	85.99%
9	14,662	739	13,923	5.04%	94.96%
10	15,420	731	14,689	4.74%	95.26%
11	14,981	1064	13,917	7.10%	92.90%
12	15,063	1042	14,021	6.91%	93.08%
13	14,518	671	13,847	4.62%	95.38%
14	14,968	1036	13,932	6.92%	93.08%
15	14,487	675	13,812	4.65%	95.34%
16	14,392	978	13,414	6.79%	93.20%
17	14,564	972	13,592	6.67%	93.33%
18	13,890	795	13,095	5.72%	94.28%
19	15,722	2182	13,540	13.87%	86.12%
20	15,349	653	14,696	4.25%	95.75%
21	15,503	952	14,551	6.14%	93.86%
22	14,953	2438	12,515	16.30%	83.70%
23	15,199	760	14,439	5.00%	95.00%
24	15,361	1060	14,301	6.90%	93.10%
25	14,236	746	13,490	5.24%	94.76%
26	15,422	689	14,733	4.46%	95.53%
27	14,949	717	14,232	4.79%	95.20%
28	15,393	647	14,746	4.20%	95.80%

29	15,393	773	14,620	5.02%	94.98%
30	15,412	1084	14,328	7.03%	92.97%
31	15,777	879	14,898	5.57%	94.43%
32	14,964	1242	13,722	8.29%	91.70%
33	15,352	687	14,665	4.47%	95.53%
34	14,532	702	13,830	4.83%	95.17%
35	15,259	891	14,368	5.83%	94.16%
36	15,059	924	14,135	6.13%	93.86%
37	15,522	827	14,695	5.32%	94.67%
38	15,545	1200	14,345	7.71%	92.28%
39	13,650	482	13,168	3.53%	96.47%
40	14,618	767	13,851	5.24%	94.75%
41	14,338	584	13,754	4.07%	95.93%
42	14,962	661	14,301	4.41%	95.58%
43	15,520	691	14,829	4.45%	95.55%
44	15,188	696	14,492	4.58%	95.42%
45	13,641	584	13,057	4.28%	95.72%
46	14,902	792	14,110	5.31%	94.69%
47	13,609	538	13,071	3.95%	96.05%
48	15,658	1082	14,576	6.91%	93.09%
49	14,648	1117	13,531	7.62%	92.37%
50	14,320	701	13,619	4.89%	95.10%
51	15,391	1366	14,025	8.87%	91.12%
52	15,902	954	14,948	5.99%	94.00%
53	15,404	743	14,661	4.82%	95.18%
54	15,621	5141	10,480	32.91%	67.09%
55	15,465	723	14,742	4.67%	95.32%
56	15,531	3619	11,912	23.30%	76.70%
57	15,665	1817	13,848	11.59%	88.40%
58	15,509	1044	14,465	6.73%	93.27%
59	13,268	546	12,722	4.11%	95.88%
60	12,905	523	12,382	4.05%	95.95%
61	14,973	642	14,331	4.28%	95.71%
62	15,658	1389	14,269	8.87%	91.13%
63	15,625	2662	12,963	17.03%	82.96%
64	12,944	766	12,178	5.91%	94.08%
65	15,235	779	14,456	5.11%	94.89%
66	15,612	1094	14,518	7.00%	92.99%
67	15,079	752	14,327	4.98%	95.01%
68	15,502	735	14,767	4.74%	95.26%
69	15,108	1052	14,056	6.96%	93.04%
70	15,704	1459	14,245	9.29%	90.71%
71	15,534	896	14,638	5.76%	94.23%
72	15,723	1155	14,568	7.34%	92.65%
73	15,755	823	14,932	5.22%	94.78%
74	15,738	797	14,941	5.06%	94.94%
75	15,752	907	14,845	5.75%	94.24%
76	15,277	825	14,452	5.40%	94.60%

77	15,375	1435	13,940	9.33%	90.67%
78	15,352	707	14,645	4.60%	95.39%
79	15,602	873	14,729	5.59%	94.40%
80	14,833	710	14,123	4.78%	95.21%
81	14,377	700	13,677	4.86%	95.13%
82	14,649	790	13,859	5.39%	94.61%
83	15,332	922	14,410	6.01%	93.99%
84	14,730	963	13,767	6.53%	93.46%
85	15,148	650	14,498	4.29%	95.71%
86	14,970	1054	13,916	7.04%	92.96%
87	15,029	843	14,186	5.60%	94.39%
88	13,929	604	13,325	4.33%	95.66%
89	14,345	1040	13,305	7.24%	92.75%
90	14,942	1974	12,968	13.21%	86.79%
91	14,151	4709	9442	33.27%	66.72%
92	14,305	666	13,639	4.65%	95.34%
93	14,032	804	13,228	5.72%	94.27%
94	14,785	844	13,941	5.70%	94.29%
95	15,155	747	14,408	4.92%	95.07%
96	15,005	712	14,293	4.74%	95.25%
97	14,909	1190	13,719	7.98%	92.02%
98	15,330	2794	12,536	18.22%	81.77%
99	15,132	754	14,378	4.98%	95.02%
100	15,144	1103	14,041	7.28%	92.72%
101	15,173	2470	12,703	16.27%	83.72%
102	15,064	778	14,286	5.16%	94.84%
103	14,376	671	13,705	4.66%	95.33%
104	15,221	703	14,518	4.61%	95.38%
105	14,256	809	13,447	5.67%	94.33%
106	15,350	837	14,513	5.45%	94.55%
107	14,578	738	13,840	5.06%	94.94%

Table S5. Analysis of QTLs for fiber quality and yield traits in three environments.

QTL	Marker	Chr.	Position (Mb)	Env.	LOD	PVE (%)	Add
qMIC-A05-1	M549	ChrA05	26.89	1	3.02	8.92	−0.36
qMIC-D03-1	M2107	ChrD03	25.57	1	4.45	13.57	−0.32
qMIC-A06-1	M593	ChrA06	69.49	2	3.35	10.04	0.25
qMIC-D011-1	M3071	ChrD11	65.79	2	34.00	12.17	0.43
qFL-A03-1	M450	ChrA03	16.10	3	6.45	6.31	0.84
qFL-A10-1	M1096	ChrA10	93.40	3	7.57	7.59	0.74
qFL-A12-1	M1189	ChrA12	66.13	3	12.87	14.59	0.63
qFL-A12-2	M1205	ChrA12	10.69	3	7.07	7.01	−0.99
qFL-A13-1	M1395	ChrA13	36.84	3	11.55	12.69	−1.20
qFL-D05-1	M2255	ChrD05	51.36	3	7.08	7.01	−0.48
qFL-D06-1	M2285	ChrD06	17.70	3	3.02	2.73	0.25
qFL-D06-2	M2552	ChrD06	50.30	3	5.81	5.59	−0.64
qFL-D10-1	M2978	ChrD10	12.29	3	4.58	4.30	−0.40
qFL-D10-2	M3030	ChrD10	61.85	3	7.49	7.50	−10.02
qFU-A06-1	M629	ChrA06	17.68	3	4.60	17.94	−1.22
qFS-D02-1	M1897	ChrD02	18.22	1	3.99	15.76	1.88
qFS-A01-1	M82	ChrA01	77.65	2	3.06	8.45	−0.96
qFS-A01-2	M253	ChrA01	85.41	2	4.43	12.64	1.81
qFS-A09-1	**M993**	**ChrA09**	**46.41**	**2**	**7.08**	**21.61**	**1.59**
qFS-A01-3	M28	ChrA01	20.44	3	3.12	9.15	−1.17
qFS-A09-2	**M993**	**ChrA09**	**46.41**	**3**	**4.54**	**13.75**	**0.97**
qFE-A08-1	M2821	ChrD08	45.58	1	3.51	10.00	0.52
qFE-A11-1	M1140	ChrA11	18.47	2	4.91	15.12	0.90
qFE-A12-1	M1204	ChrA12	10.27	2	3.16	9.32	0.71
qFE-D06-1	M2339	ChrD06	28.90	2	4.57	13.94	0.67
qFE-A02-1	M345	ChrA02	12.27	3	5.16	9.92	0.30
qFE-A13-1	M1309	ChrA13	13.99	3	4.64	8.81	−0.23
qFE-D02-1	M1978	ChrD02	22.44	3	6.63	13.17	0.24
qFE-D02-2	M2057	ChrD02	56.73	3	4.62	8.78	0.44
qFE-D07-1	M2816	ChrD07	44.50	3	4.74	9.02	0.29
qBW-D04-1	M2170	ChrD04	40.54	1	3.05	13.33	0.20
qBW-A07-1	M749	ChrA07	68.67	2	6.83	19.53	−0.62
qBW-A09-1	M900	ChrA09	45.81	2	3.23	8.44	0.16
qBW-A09-2	M1027	ChrA09	66.34	2	24.26	11.42	0.41
qBW-D10-1	M3016	ChrD10	56.48	2	3.17	8.25	−0.32
qBW-A03-1	M436	ChrA03	15.68	3	3.90	10.97	−0.26
qBW-A13-1	M1458	ChrA13	40.74	3	4.17	11.83	−0.33
qLW-A07-1	M749	ChrA07	68.67	2	3.43	10.46	−0.22
qLW-D10-1	M3016	ChrD10	56.48	2	5.15	16.41	−0.21
qLW-A05-1	M561	ChrA05	35.06	3	4.24	15.97	−0.43
qLP-A07-1	**M691**	**ChrA07**	**10.97**	**1**	**7.71**	**25.95**	**−6.36**
qLP-A01-1	M77	ChrA01	77.52	3	7.19	4.25	−0.78
qLP-A01-2	M227	ChrA01	84.35	3	8.53	5.20	1.60
qLP-A05-1	M561	ChrA05	35.06	3	29.58	30.15	−10.53
qLP-A07-2	**M695**	**ChrA07**	**11.15**	**3**	**10.98**	**7.08**	**5.10**

qLP-A11-1	M1133	ChrA11	15.17	3	12.61	8.44	5.57
qLP-A11-2	M1158	ChrA11	19.11	3	7.05	4.15	−1.98
qLP-A12-1	M1273	ChrA12	77.44	3	3.70	2.02	1.24
qLP-A13-1	M1575	ChrA13	48.09	3	5.19	2.93	2.33
qLP-A13-2	M1800	ChrA13	70.58	3	8.23	4.98	−3.04
qLP-D02-1	M1955	ChrD02	20.99	3	6.16	3.56	0.71
qLP-D05-1	M2216	ChrD05	20.31	3	5.69	3.26	1.20
qLP-D06-1	M2580	ChrD06	51.28	3	11.92	7.85	−2.25
qHSW-A02-1	M357	ChrA02	40.7	2	10.74	11.42	1.08
qHSW-A03-1	M421	ChrA03	45.13	2	7.48	7.38	0.63
qHSW-A06-1	M670	ChrA06	10.18	2	4.90	4.56	0.79
qHSW-A09-1	M934	ChrA09	93.82	2	8.29	8.33	1.30
qHSW-A13-1	M1567	ChrA13	47.99	2	3.15	2.82	−0.75
qHSW-A13-2	M1686	ChrA13	54.02	2	15.09	17.77	1.04
qHSW-A13-3	M1721	ChrA13	58.85	2	4.83	4.49	0.68
qHSW-D02-1	M2014	ChrD02	37.78	2	5.14	4.82	−0.48
qHSW-D06-1	M2286	ChrD06	17.7	2	3.33	3.00	−0.22
qHSW-D06-2	M2461	ChrD06	38.2	2	8.86	9.02	0.47
qHSW-D07-1	M2793	ChrD07	31.31	2	4.04	3.69	0.62
qPH-D06-1	M2529	ChrD06	46.57	1	3.28	11.44	−5.52
qPH-D11-1	M3045	ChrD11	53.39	1	4.47	16.08	9.09
qPH-A13-1	M1620	ChrA13	49.16	2	3.13	10.93	7.35
qBN-A1-1	M305	ChrA01	86.96	1	3.26	14.76	8.55
qBN-A13-1	M1483	ChrA13	41.87	2	3.32	7.74	−7.02
qBN-A13-2	M1792	ChrA13	68.22	2	3.45	8.05	11.17
qBN-D02-1	M2034	ChrD02	44.45	2	6.27	15.61	9.98
qFBN-D02-1	M1926	ChrD02	19.37	3	3.02	13.07	1.06
qSI-A02-1	M357	ChrA02	40.7	3	3.62	10.03	0.66
qSI-A13-1	M1686	ChrA13	54.02	3	6.17	18.10	0.68

MIC = micronaire, FL = fiber length, FU = fiber uniformity, FS = fiber strength, FE = fiber elongation, BW = boll weight, LW = lint weight, LP = lint percentage, HSW = hundred seed weight, PH = plant height, BN = boll number, FBN = fruiting branches number, SI = seed index, Chr. = Chromosome, Env. = Environment, LOD = Logarithm of odds, PVE = phenotypic variance explained, Add. Additive effects. The QTLs are highlighted as bold are stable QTLs.

A

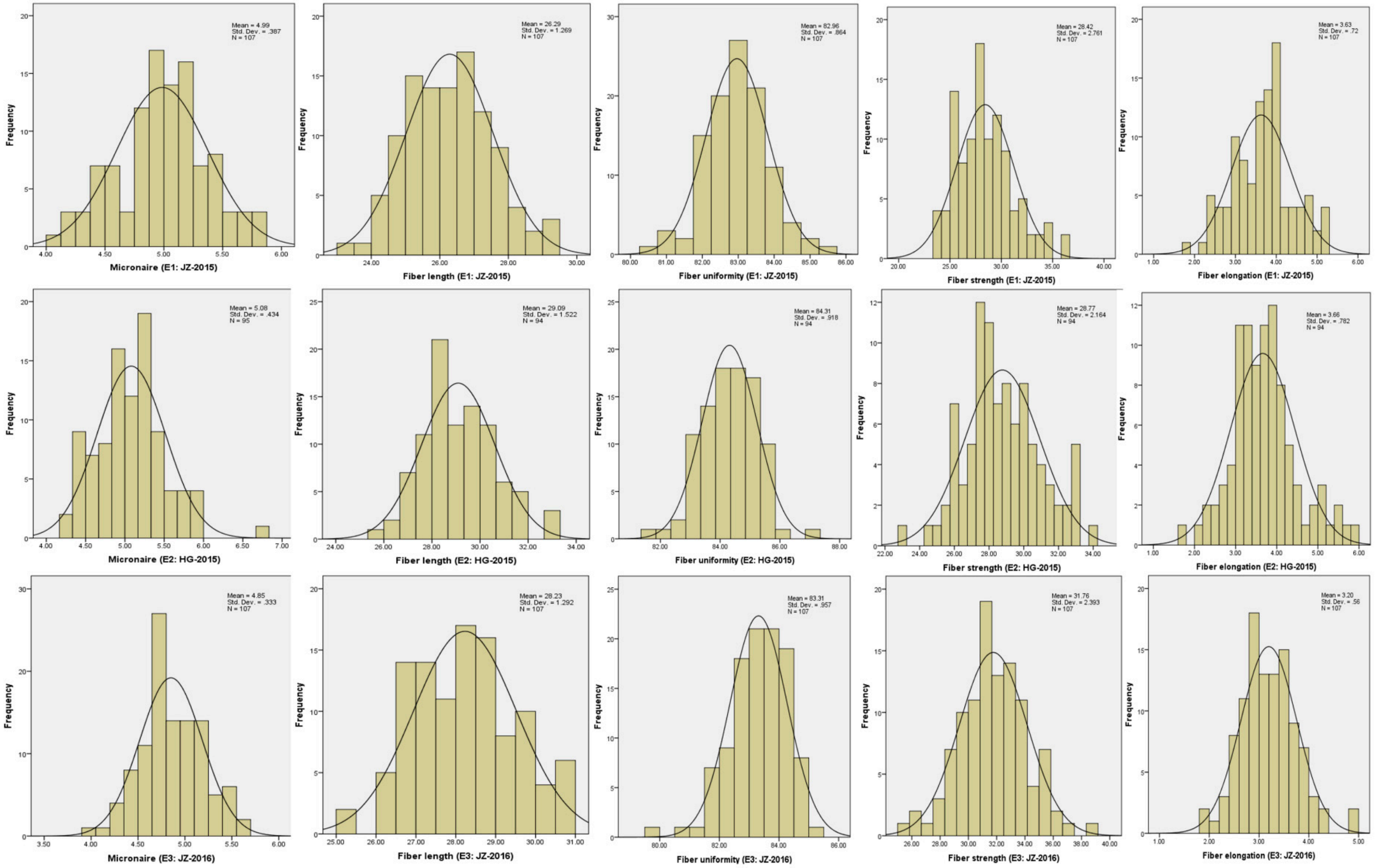
Mean = 4.99
Std. Dev. = .387
N = 107
Frequency
Micronaire (E1: JZ-2015)
Mean = 26.29
Std. Dev. = 1.269
N = 107
Frequency
Fiber length (E1: JZ-2015)
Mean = 82.96
Std. Dev. = .864
N = 107
Frequency
Fiber uniformity (E1: JZ-2015)
Mean = 28.42
Std. Dev. = 2.761
N = 107
Frequency
Fiber strength (E1: JZ-2015)
Mean = 3.63
Std. Dev. = .72
N = 107
Frequency
Fiber elongation (E1: JZ-2015)
Mean = 5.08
Std. Dev. = .434
N = 95
Frequency
Micronaire (E2: HG-2015)
Mean = 29.09
Std. Dev. = 1.522
N = 94
Frequency
Fiber length (E2: HG-2015)
Mean = 84.31
Std. Dev. = .918
N = 94
Frequency
Fiber uniformity (E2: HG-2015)
Mean = 28.77
Std. Dev. = 2.164
N = 94
Frequency
Fiber strength (E2: HG-2015)
Mean = 3.66
Std. Dev. = .782
N = 94
Frequency
Fiber elongation (E2: HG-2015)
Mean = 4.85
Std. Dev. = .333
N = 107
Frequency
Micronaire (E3: JZ-2016)
Mean = 28.23
Std. Dev. = 1.292
N = 107
Frequency
Fiber length (E3: JZ-2016)
Mean = 83.31
Std. Dev. = .957
N = 107
Frequency
Fiber uniformity (E3: JZ-2016)
Mean = 31.76
Std. Dev. = 2.393
N = 107
Frequency
Fiber strength (E3: JZ-2016)
Mean = 3.20
Std. Dev. = .56
N = 107
Frequency
Fiber elongation (E3: JZ-2016)

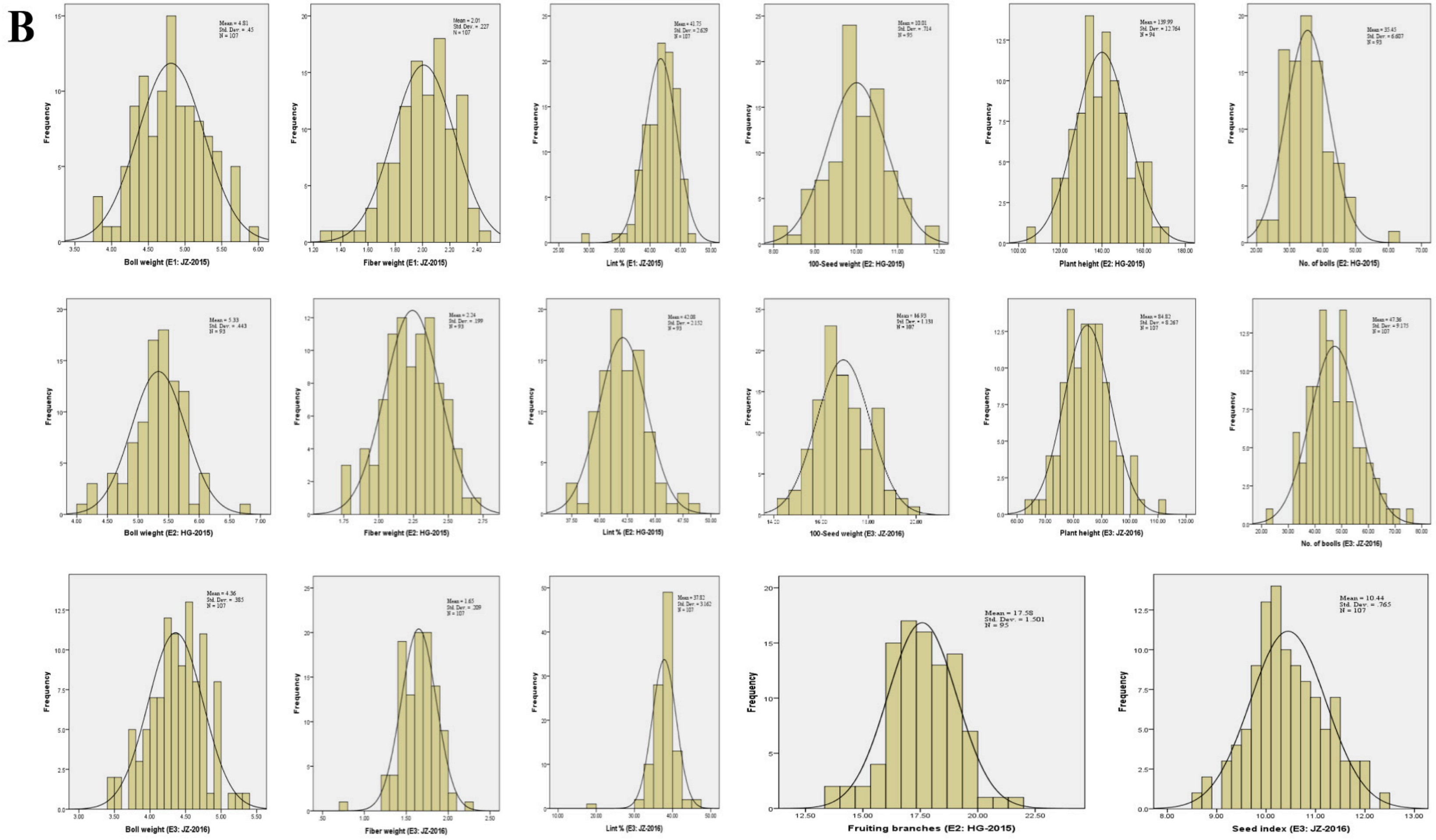

Figure S1. Phenotypic distribution of (**A**) five fiber quality traits and (**B**) eight yield traits in the ILs across three environments.

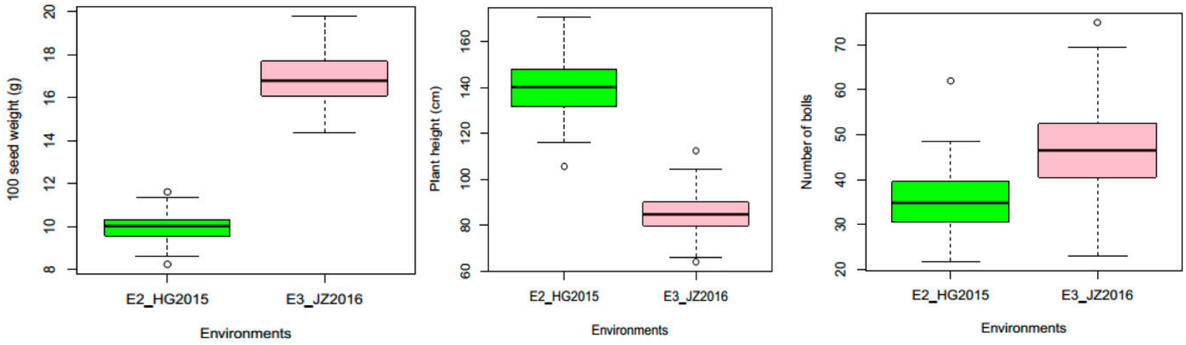

Figure S2. Box plot of the changing trends of three yield traits in two environments (FBN and SI only in E3 were not analyzed).

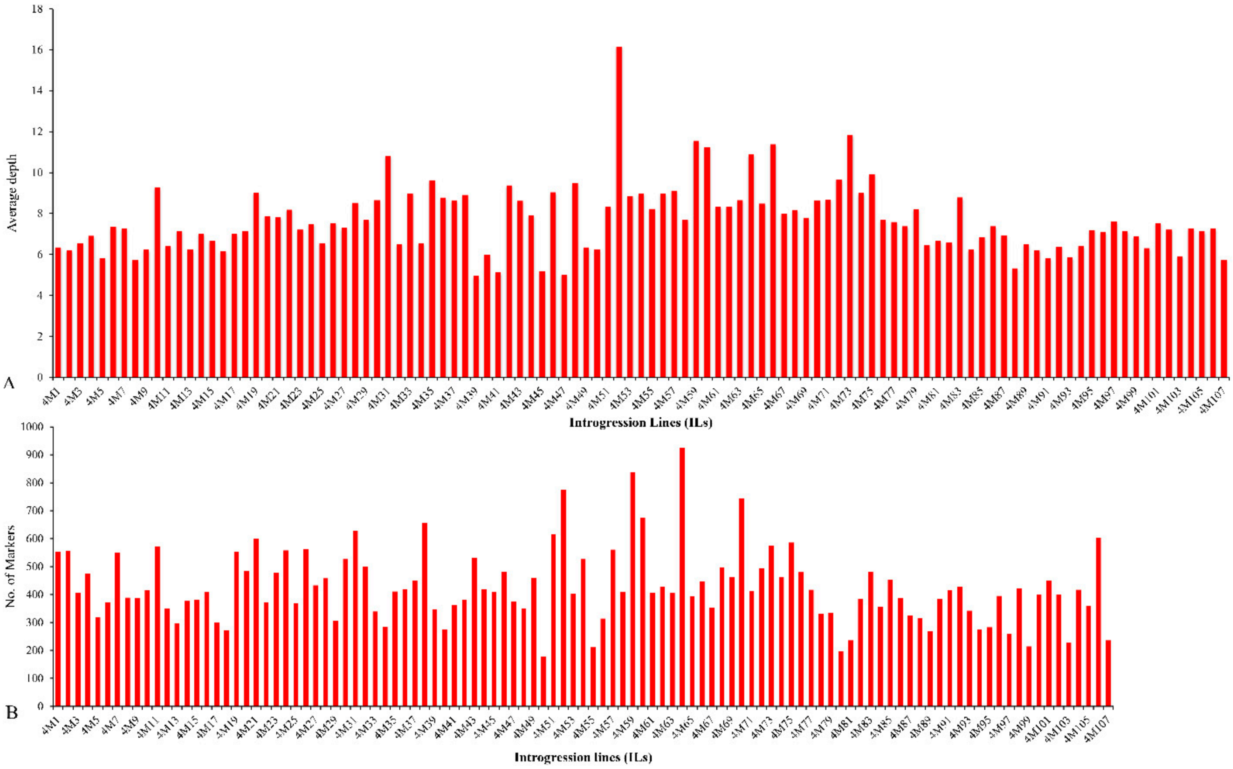

Figure S3. The x-axes in (**A**) and (**B**) indicate introgression lines, and the y-axes indicate the average sequencing depth (a) and number of markers (b).
